# Immigrant Parents’ Knowledge and Attitudes: Sociodemographic Variation Related to Restriction of Children’s Sugar Intake

**DOI:** 10.3290/j.ohpd.b5816441

**Published:** 2024-11-07

**Authors:** Manal Mustafa, Elwalid Nasir, Abhijit Sen, Anne Nordrehaug Åstrøm

**Affiliations:** a Senior Researcher, Oral Health Centre of Expertise in Western Norway, Vestland, Bergen, Norway. Study concept, methodology, validation, investigation, data collection and analysis, wrote original draft, project administration.; b Associate Professor, King Faisal University SA, University of Science and Technology, Omdurman, Sudan. Methodology, validation, analysis, reviewed and edited the manuscript.; c Associate Professor, Oral Health Services and Research (TkMidt), Norwegian University of Science and Technology, Trondheim, Norway. Methodology, validation, reviewed and edited the manuscript.; d Professor, Department of Clinical Dentistry, Faculty of Medicine, University of Bergen, Bergen, Norway; Oral Health Centre of Expertise in Western Norway, Vestland, Bergen, Norway. Methodology, validation, formal analysis, data curation, reviewed and edited the manuscript.

**Keywords:** attitude, children, immigrant, knowledge, sugar consumption

## Abstract

**Purpose:**

Focusing on immigrant parents with children aged 0–6 months, this study assessed whether awareness of and attitudes towards restricting children’s sugar consumption vary according to family sociodemographic background and parents’ oral-health–related behaviours.

**Materials and Methods:**

A cross-sectional study was conducted including immigrant parents attending child public health centers for vaccination of their children. The study included parents born in Asia, Africa, South America, Central America and Eastern Europe. Parents from Western Europe and North America were included if they were partners of the above-mentioned participants. Cross-tabulation and multiple variable logistic regression were used to assess associations of parental knowledge and attitudes with their child’s sugar intake and sociodemographic characteristics.

**Results:**

Overall, response rate was 72.6%. A total of 345 parents completed personal, structured interviews during their visit to the health centers. Attitudes and knowledge, but not indulgence, related to children’s sugar restriction were sociodemographically unequally distributed among immigrant parents. Employed mothers, mothers with immigrant background from North America or Western Europe as well as parents with less frequent own sugar intake were more likely to confirm positive attitudes towards restricting children’s sugar snacking. The corresponding odds ratios were OR=1.8 (95% CI 1.1–3.1) and OR=6.6 (95% CI 2.3–18.9). Employed mothers and parents having received dental care information were more likely than their counterparts to possess good oral-health–related knowledge.

**Conclusion:**

Parents from sociodemographically disadvantaged backgrounds were less inclined to express positive attitudes and demonstrate sufficient knowledge regarding the limitation of their children’s sugar snacking. Culturally adapted oral health intervention programs should be implemented for immigrants, with special reference to children’s dietary habits.

Early Childhood Caries (ECC), the most common chronic disease affecting infants and young children, has a skewed distribution in most industrialised countries.^1,32^ A conceptual model illustrates the multifactorial aetiology of ECC in terms of influences at child-, family- and environmental levels, indicating indirect and direct impacts on its development.^19,23^ At the child level, frequent consumption of cariogenic carbohydrates for longer periods has been reported to increase the risk of ECC.^30,35^ At the family and environmental level, risk indicators include parental socioeconomic status, such as education, family income, and cultural background, as well as parental lifestyles, in addition to their knowledge of and attitudes towards caring for their children’s teeth.^30^ It is evident that parental oral-health–related behaviours influence early establishment of the corresponding habits in their children, implying that early childhood constitutes an important period for future oral health.^8,26^ A Norwegian cohort study revealed that children having a mother of non-Western origin with lower education and who consumed a diet containing more fat or sugar than recommended were at increased risk of having caries experience at the age of 5 years.^37^

In Scandinavia, research has shown overall poorer health and oral health status in non-Western immigrant children than in their ethnic majority counterparts.^27,32^ It is evident that parents from countries outside Europe and parents who immigrated after the age of 20 years were two to four times more likely to have a child with ECC than European parents or parents who immigrated at a younger age.^36^ Immigrants also tend to be more socioeconomically disadvantaged and suffer more from diet-related disorders in general compared to the native populations.^27^ Among adult immigrants, the transition from a whole grain diet rich in fiber, to a diet high in fat and sugar, including fewer vegetables, contributes to a higher risk for diabetes and cardiovascular diseases as well as to obesity and dental caries in their children.^9,18^

Changes in dietary behaviour is attributable to diverse factors, including language difficulties, lack of acculturation, shortage of previously used products, and culturally related factors.^11,21^ Evidence suggests that oral health illiteracy and poor knowledge in mothers from minority groups is associated with poor oral health outcomes and less frequent use of oral health care services among their children.^32^ A previous study revealed that mothers with less knowledge about detrimental oral health behaviours, for instance, the belief that dental caries is not triggered by using infant drinking bottle containing fluids other than water, were more likely to have infants consuming a high frequency of free sugars.^4,6,21^ According to social cognition theory, parents’ positive attitudes towards children’s sugar-consumption control constitutes an important determinant of their oral-health–promoting feeding behaviours.^2^ This has been confirmed in previous studies considering restriction of sugar intake in Ugandan preschool children^3^ and UK mothers’ decision to limit frequency of infants’ sugar intake.^5^

Although the evidence of risk indicators of ECC at environmental, family and child levels is substantial, there is a paucity of such information from subgroups of immigrant parents and their children under 3 years of age. Information about how immigrant parents’ socio-behavioural characteristics correlate with their knowledge and attitudes related to sugar restriction in infants and younger children have important implications for the implementation of dental health programs. Such information should be available before clinical caries is visible in children to facilitate preventive strategies and early contact with dental healthcare services.

Previous studies have demonstrated that the frequency of sugar consumption is higher than recommended in children even in the first year of life.^13,24^ Thus, restricting frequent consumption of added non-milk extrinsic sugars remains a justifiable part of caries prevention for immigrant children. This study focuses on immigrants in Norway with children aged 0-6 months. The hypothesis of the present study suggests a sociodemographic variation of immigrant parents’ knowledge and attitudes related to restriction of children’s sugar intake. Therefore, the aim was to assess immigrant parents’ level of oral-health–related knowledge, attitudes, and indulgence related to children’s sugar-consumption restriction and to identify how those concepts vary according to parental sociodemographic background and their own sugar consumption.

## MATERIALS AND METHODS

Immigrant parents of foreign background were recruited from public primary healthcare child service centers in Bergen when they attended for children’s vaccination. The required sample size was estimated to be 300 parent/child pairs by using Satterthwaite’s t-test assuming unequal variance between individuals having satisfactory rather than unsatisfactory oral-health–related knowledge. By taking into consideration the possibility of non-response, extra participants were recruited. The recruitment process has been described in detail in a previous paper.^28^ Briefly, the participants were immigrants – either mother, father or both –born outside Norway. In this study parents born in Asia, Africa, South America, Central America and Eastern Europe were included. Parents from Western Europe and North America were included if they were partners of the above-mentioned participants. In this study, all immigrant parents who attended the primary health care centers during the period of the study, who gave consent and agreed to follow the study procedures, were invited to participate. A professional researcher explained the purpose and conducted a face-to-face interview while parents and children waited out the recommended time after vaccination before discharge. The interview was conducted in Norwegian, and interpreters were used for those with limited Norwegian skills. The interview lasted between 15-20 min. Participation was voluntary and written consent was obtained from all participants. The study received ethical approval from the Regional Committee for Medical Research Ethics (2015/2037/REK vest).

### Measures

Sociodemographic characteristics were assessed in terms of parental age, education (lower, higher level), length of fathers’ residence in Norway (shorter: 1-8 years; longer: < 8 years), length of mothers’ residence in Norway (shorter: 1–6 years; longer: < 6 years), geographic origin (Asia/Africa, Eastern Europe/South America, North America/Western Europe) and employment status (employed, not employed).

Attitudes towards control of children’s sugar consumption were assessed by a sum score of the following items: (1) as a mother/father I can easily control children’s sugar intake between meals; (2) As a mother/father, it is difficult to control children’s frequent sugar intake. (3) As mother/father; I want to control children’s sugar intake; (4) It makes sense to control children’s sugar intake; (5) In our family we think it is unfair not to give children sugared snacks every day; (6) My family consider it important to control children’s sugar intake. The items were assessed using a Likert scale ranging from (1) totally agree to (5) totally disagree. Before adding the items together, response scales of the negatively worded items were reversed. A sum score of attitudes towards sugar restriction was constructed, ranging from 6 (positive) to 20 (negative) and dichotomized on a medium split into positive attitudes (coded as 1) negative attitudes towards children’s sugar control (coded as 0).

Knowledge of children’s oral health was assessed by a sum score of ten items, e.g., “Dental caries is commonly seen in children” and “High intake of sugary snacks such as chocolate can lead to caries”, “High intake of drinks containing sugar such as cola cannot lead to caries”, “Frequent night breastfeeding of the child can lead to caries”. Response categories used were correct (1) and not correct/I do not know (0). A few questions were negatively worded to check the participants’ understanding of the questions. A sum score of oral-health–related knowledge was constructed after reversing the negative items (range: 8 [good] to 18 [poor]). The sum score was dichotomised on a median split into “good knowledge” (1) and “poor knowledge” (0).

Parents own sugar consumption was assessed by a sum score of two items “How often do you eat sugary snacks?” and “How often do you drink drinks with added sugar?”. Each item was assessed on a frequency scale ranging from “every day or almost every day” (1) to “never” (5). A sum score was constructed by adding the items and then dichotomised on a median split into “infrequent sugar intake” (1) and “frequent sugar intake” (0).

Parental indulgence was assessed by one item: “It is often stressful to deny children sugared snack when they request this” assessed on a scale ranging from “totally agree” (1) to “totally disagree” (5). The score was dichotomised on a median split into “strong indulgence” (1) and “weak indulgence” (0).

Information from a dentist on how to taking care of children’s teeth was assessed by one question and replied to in terms of “no” (0) and “yes” (1).

### Statistical Methods

Data were analysed using IBM SPSS Statistics for Windows version 25 (IBM; Armonk, NY, USA). Descriptive statistics were performed using mean and standard deviation (SD) for count and continuous variables and percentages for categorical variables. Unadjusted analyses were performed using cross tabulation and the chi-squared test. Multiple variable logistic regression analyses were conducted to assess the association of sociodemographic factors, parents’ own sugar intake, and received information from dentist, with parental knowledge, attitudes and indulgence related to children’s sugar restriction using odds ratios (OR) and corresponding 95% confidence intervals (CI).

## RESULTS

A total of 345 immigrant parents (response rate 72.6%) completed personal interviews at healthcare centers in Bergen. The mean age of the mothers and fathers were (30.4±5.2 years) and (35.4±6.9. years), respectively.

As depicted in Table 1, more than half of the participating mothers and fathers belonged to younger age groups, and 64.1% of participating mothers and 61.7% of participating fathers were immigrants from Africa/Asia. Corresponding percentages of mothers and fathers from Eastern Europe/South America were 23.5% and 16.9%, and North America/Western Europe were 12.5 % and 21.4%, respectively. Higher proportions of fathers than mothers had longer residence stays in Norway (60.3% vs 47.0%) and about half of both mothers and fathers had received a higher education (university or college). A total of 66.4% and 40.6% of parents reported frequent own intake of sugared snacks and drinks and no information received from the dentist, respectively.

**Table 1:** Immigrant parents’ sociobehavioural characteristics (n = 345)

Characteristic	% (n)
**Mother’s age**	
Younger (18–30)	62.4 (179)
Older (>30)	37.6 (108)
**Father’s age**	
Younger (22–35)	55.3 (182)
Older (>35)	44.7 (147)
**Mother’s education**	
Lower	44.3 (153)
Higher	55.7 (192)
**Father’s education**	
Lower	46.8 (159)
Higher	53.2 (181)
**Mother’s length of stay in Norway**	
Shorter: 1–6 years	53.0 (183)
Longer: >6 years	47.0 (162
**Father’s length of stay in Norway**	
Shorter: 1–8 years	39.7 (137)
Longer: >8 years	60.3 (208)
**Migration background mother**	
Asia/Africa	64.1 (221)
Eastern Europe/South America	23.5 (81)
North America/Western Europe	12.5 (43)
**Migration background father**	
Asia/Africa	61.7 (208)
Eastern Europe/South America	16.9 (57)
North America/Western Europe	21.4 (72)
**Mother employed**	
Yes	57.0 (196)
No	43.0 (148)
**Father employed**	
Yes	82.2 (268)
No	17.8 )58)
**Parent sugar score**	
Negative (frequent)	66.4 (229))
Positive (infrequent)	33.6 (116)
**Information obtained from dentist**	
No	40.6 (140)
Yes	59.4 (205)


As shown in Table 2, the proportion of fathers from Asian/African, Eastern European and North American/Western European countries reporting frequent sugar consumption amounted to 69.2%, 66.7% and 59.7%, while among mothers the proportions were 68.3%, 60.5% and 67.4%. Sweetened tea and coffee were the most common sugary products reported (data not shown).

**Table 2:** Immigrant parents’ sugar consumption by cultural background (n = 345)

Sugar consumption	Asia/Africa % (n)	Eastern Europe % (n)	North America % (n)
Fathers Frequent	69.2 (144)	66.7 (38)	59.7 (43)
Infrequent	30.8 (64)	33.3 (19)	40.3 (29)
Mothers Frequent	68.3 (151)	60.5 (49)	67.4 (29)
Infrequent	31.7 (70)	39.5 (32)	32.6 (14)


As shown in Table 3, percentages of participants that responded correctly to single knowledge statements regarding children’s oral health ranged from 25.7% (sugar should be consumed during meals) to 96.8% (high sugar intake gives tooth decay). Table 4 depicts the mean sum scores of parental knowledge, attitudes and indulgence related to children’s sugar restriction. As shown, immigrant parents had on average neutral to positive attitudes towards children’s sugar restriction, average to good knowledge of how to care for children’s teeth and neutral indulgence regarding children’s sugar control. Totals of 50.0%, 54.6% and 29.9% had good knowledge, positive attitudes towards sugar control and strong indulgence, respectively, regarding sugar restriction among their children (not shown in the table).

**Table 3:** Parental knowledge towards restricting child’s sugar intake

Knowledge items	Correct % (n)	Not correct/don’t know % (n)
Tooth decay is common in children	35.7 (123)	64.3 (222)
High sugar intake causes tooth decay	96.8 (334)	3.2 (11)
Sugared drinks cannot cause tooth decay	88.7 (306)	11.3 (39)
Breastfeeding during the night can cause tooth decay	29.7 102)	70.3 (242)
Sugar-containing food should be eaten during meals	25.7 (88)	74.3 (255)
Sugar-containing food should be eather between meals	47.1 (162)	52.9 (182)
Start toothbrushing when first tooth has erupted	80.6 (275)	19.4 (66)
Start toothbrushing when all primary teeth have erupted	81.2 (280)	18.8 (65)
Toothbrushing twice a day prevent tooth decay	86.0 (296)	14.0 (48)
Regular use of fluoridated toothpaste can cause tooth decay	69.8 (240)	30.2 (104)


**Table 4:** Mean (SD) of sum scores of knowledge, attitudes and indulgence (n = 345)

	Mean (SD)	Range (theoretical range)
Sum knowledge	13.6 (1.6)	10–18 (good-poor)
Sum attitudes	10.6 (3.2)	6–20 (positive-negative)
Indulgence	3.5 (1.3)	1–5 (strong-weak)
		

Table 5 depicts the unadjusted and adjusted associations of parents’ socio-behavioural characteristics with attitudes and indulgence regarding restriction of children’s sugar intake. Unadjusted cross tabulation revealed that higher proportions of highly educated mothers and fathers, fathers and mothers with longer residence in Norway, and employed mothers reported positive attitudes towards children’s sugar restriction compared to their counterparts in the opposite groups. A similar positive attitude was revealed among fathers, and mothers with immigrant background from North America/Western Europe. In addition, higher proportions of parents with infrequent own sugar consumption and who confirmed dental care information from dentist reported positive attitudes. Parental indulgence was least frequent in fathers and mothers with immigrant background from Eastern/Western Europe and North America, as well as in parents who confirm receiving information from dentist.

**Table 5:** Parents’ attitudinal and indulgence perceptions regressed on mother and fahters’ sociodemographic characteristics and own oral-health–related behaviours, percentages who confirm positive attitudinal and indulgence perceptions

Independent variable	Positive attitude		Indulgence high	
	% (n)	OR (95% CI)	% (n)	OR (95% CI)
**Sociodemographics**				
Father’s age (22–35)	50.0 (88)		29.7 (54)	
Father’s age >35	58.6 (85)		27.9 (41)	
Mother’s age ≤30	50.6 (88)		26.3 (47)	
Mother’s age <30	61.3 (65)		34.3 (37))	
**Father’s education**				
Lower	52.9 (82)		27.0 (43)	
Higher	57.6 (102)		32.6 (59)	
**Mother’s education**				
Lower	45.6 (67)	1	26.8 (41)	
Higher	61.6 (117)*	1.44 (0.8–2.3)	32.3 (62)	
**Father’s length of stay**				
Shorter	44.0 (59)	1	32,1 (44)	
Longer	61.6 (125)*	1.5 (0.8–2.5)	28.4 (59)	
**Mother’s length of stay**				
Shorter	45.8 (82)	1	32.1 (44)	
Longer	64.(102)**	1.2 (0.7–2.2)	28.4 (59)	
**Father employed**				
No	43.6 (24)	1	22.4 (13)	
Yes	56.4 (149)	2.1 (0.8–5.5)	30.6 (82)	
**Mother employed**				
No	41.4 (60)	1	25.7 (38)	
Yes	64.4(123)**	1.8 (1.1–3.1)	33.2 (65)	
**Immigrant background of father**				
Asia/Africa	48.8 (98))	1	31.3 (65)	
Eastern Europe	54.4 (31)	0.7 (0.3–2.2)	19.3 (11)	
North America/Western Europe	70.4 (50)**	1.7 (0.8–3.6)	30.6 (22)	
**Immigrant background of mother**				
Asia/Africa	46.3 (100)	1	34.4 (76))	1
Eastern Europe	59.5 (47)	1.4 (0.5–3.6)	22.1 (18)	0.5 (0.3–1.0)
North America/Western Europe	88.1 (37)**	6.6 (2.3–18.9)	20.9 (9)*	0.5 (0.2–1.2)
**Parental sugar consumption**				
Frequent	50.7 (113)	1	31.9 (73)	
Infrequent	62.3 (71)*	1.6 (1.0–2.5)	25.9 (30)	
**Information from a dentist**				
No	45.6 (62)	1	35.0 (49)	1
Yes	60.7 (122)*	1.2 (0.7–1.9)	26.3 (54)*	0.7 (0.4–1.2)

Adjusted logistic regression analysis. Odds ratios (OR) and 95% confidence interval (CI).

Adjusted logistic regression analyses, including socio-behavioural variables that were statistically significantly associated with attitudes and indulgence in unadjusted cross tabulation analyses, revealed that employed mothers, mothers with immigrant background from North America/Western Europe and parents with less frequent own sugar consumption were most likely to have a positive attitude towards children’s sugar restriction. The corresponding odds ratios were OR=1.8 (95% CI 1.1–3.1), OR=6.6 (95% CI 2.3–18.9) and OR=1.6 (95% CI 1.0–2.5). No sociodemographic variable remained statistically significant for indulgence in adjusted logistic regression analysis.

As shown in Table 6, according to unadjusted analyses, mothers and fathers with higher education, with longer residence stay in Norway, employed mothers, and parents having acquired information from dentist were more likely to possess good knowledge about caring for children’s teeth than their counterparts in the opposite groups. Adjusted logistic regression, adjusting for socio-behavioural variables that were statistically significantly associated with knowledge in unadjusted analyses, revealed that compared to immigrant fathers from Asia/Africa, fathers from Eastern Europe were more likely to have good oral-health–related knowledge. Parents having received information regarding dental care were more likely than their counterparts to possess good knowledge. Corresponding odds ratios were OR=2.5 (95% CI 1.0–6.8) and 2.1 (95% CI 1.3–3.4). Mothers who had been in Norway longer were more likely than their counterparts with shorter stays to have good knowledge (OR=1.7 [95% CI 1.0–2.9]).

**Table 6:** Parents’ knowledge and child’s oral health risk regressed on sociodemographic characteristics and parental oral-health–related behaviours

Independent variable	Knowledge % (n)	OR (95% CI)
**Sociodemographics**		
Father’s age (22–35)	47.2 (84)	
Father’s age >35	52.4 (76)	
Mother’s age younger	45.1 (79)	
Mother’s age older	55.6 (60)	
**Father’s education**		
Lower	44.5 (69)	1
Higher	55.6 (99)*	1.8 (1.1–3.1)
**Mother’s education**		
Lower	44.3 (66)	1
Higher	54.5 (103)*	1.1 (0.6–1.9)
**Father’s length of stay**		
Shorter	43.3 (58)	1
Longer	54.4 (111)*	1.2 (0.7–2.2)
**Mother’s length of stay**		
Shorter	42.5 (77)	1
Longer	58.6 (92)**	1.7 (1.0–2.9)
**Father employed**		
No	41.8 (23)	
Yes	51.5 (136)	
**Mother employed**		
No	41.7 (60)	1
Yes	56.0 (108)*	1.1 (0.6–1.8)
**Immigrant background of father**		
Asia/Africa	42.9 (87)	1
Eastern Europe	63.2 (36)	2.5 (1.0–6.8)
North America/Western Europe	60.0 (42)*	1.4 (0.7–2.8)
**Immigrant background of mother**		
Asia/Africa	44.9 (97)	1
Eastern Europe	62.5 (50)	0.9 (0.4–2.3)
North America/Western Europe	52.4 (22)*	0.7 (0.3–1.5)
**Parental sugar consumption**		
Frequent	48.7 (109)	
Infrequent	52.6 (60)	
**Information from dentist**		
No	38.1 (53)	1
Yes	58.3 (116)**	2.1 (1.3–3.4)

Percentages of parents reporting good knowledge % (n) and adjusted odds ratio (OR) and 95% confidence interval (CI).

## DISCUSSION

Parents’ feeding practice during infancy and early childhood is an important contributor to children’s oral health trajectory because of caregivers’ feeding responsibility and since children’s preferences for food are established during early years.^26^ The present study adds to the literature regarding infants’ and younger children’s feeding habits by assessing the sociodemographic and -behavioural covariates of immigrant parents’ knowledge, attitudes and indulgence related to sugar-consumption control of their children 0-6 months of age. Immigrant parents had on average neutral to positive attitudes, average to good knowledge and neutral attitude on weak indulgence regarding control of children’s sugar intake. Moreover, the likelihood of having positive attitudes, high indulgence and good knowledge varied according to immigrant parents’ sociodemographic characteristics and their own sugar consumption. Overall, the greatest impact was that of the mothers, thus supporting evidence that mothers spend significantly more time in direct interaction with their children than do the fathers.^29^

Affluent immigrant parents, those with longer residence stay in Norway, employed mothers and mothers with North American/Western European background were more likely to score positive attitudes towards children’s sugar consumption control than other categories. An earlier study from Canada reported that marginalised immigrant parents were more likely to exhibit unfavourable oral health behaviours.^10^ Similar findings were described in a previous study from Uganda, where the most socio-economically deprived families reported less positive attitudes and motivation towards control of preschool children’s sugar consumption.^25^ Associations between higher educational level and positive dental attitudes have been documented previously among native Norwegian as well as immigrant parents.^33^ Where this study suggests that greater acculturation (i.e., longer stay of residence in Norway) promotes parents’ positive attitudes towards sugar restriction, a Canadian study found that immigrant parents’ acculturation was associated with increased sugar consumption among their children.^10^ As shown in Table 4, independent of their cultural background, parents reporting infrequent own sugar consumption were more likely to report positive attitudes towards restricting the sugar content of their feeding practice. This association was statistically significant, suggesting that consumption of sugared snacks and drinks is clustered within immigrant families. In line with the present findings, recent research from the US has shown that greater negativity in parental attitudes towards sugar sweetened beverages (SSB) is associated with less parental intake of SSB and less likelihood of infants’ SSB consumption.^38^ Sweet beverages are one of the main sources of added sugars. and parental preferences and availability of sugary items in the home are strong predictors of the children’s sugar intake.^7^ A previous study of African-American children reported strong associations between the child and parental sweet beverage intake.^16^

According to the parental feeding style framework developed by Hughes et al,^20^ indulgent parental feeding style is associated with unhealthy dietary patterns and increased likelihood of children experiencing poor oral health during their life course.^15,17^ Immigrant mothers from North America/Western Europe and parents who had not received oral health information from a dentist were less and more likely, respectively, to be indulgent regarding children’ sugar intake restriction. Other studies have shown that higher parental literacy and stress were associated with weaker and stronger indulgence, respectively.^17^ Mothers with immigrant background from Asia/Africa might seek to reward and motivate children with sweetened snacks not as part of their parental style but as a response to their child’s need and behaviour. Notably, excessive restriction of children’s sugar intake might also have counterintuitive consequences in terms of leading to increased desire for sugary food items and a higher sugar intake over time.^12^

About half of immigrant parents investigated in this study demonstrated good knowledge of children’s oral-health–promoting behaviours. However, confusion existed regarding whether sugared snacks and drinks should be taken at or between meals as well as about the risk of caries associated with nighttime breastfeeding. Mothers with a longer stay of residence in Norway, fathers with immigrant background from Eastern Europe and parents having received professional oral health information were more likely than their counterparts to present with good oral-health–related knowledge. Immigrant parents’ illiteracy has been demonstrated previously, whereby parents with immigrant background routinely gave their toddlers a bedtime bottle and were unable to control children’s sugar consumption.^14^ A previous study revealed that well-informed caregivers were motivated to control children’s consumption of sweetened snacks, but the main barrier was identified as other family members allowing consumption of sugar snacks/drinks.^22^

An important strength of this study is the use of socio-cognitive theory as a guide for data analysis and interpretation. According to the present findings, precursors of immigrant parents’ feeding practices in terms of attitudes and indulgence towards sugar restriction and oral-health–related knowledge seem to be sociodemographically distributed in a way similar to that of children’s caries experience. This agrees with theoretical assumptions that immigrant parents’ knowledge, attitudes and indulgence are important mediators of sociodemographic influences on feeding practice and children’s caries experience.^31^ Other strengths of this study are the unique focus on immigrant parents, the inclusion of both mothers’ and fathers’ sociodemographic characteristics, as well as its cross-cultural approach in terms of including parents from various parts of the world. In addition, attitudinal and indulgence beliefs utilised here have been used previously, and demonstrated to be related to caries experience and to be valid in a longitudinal perspective.^33,34^ Sampling of healthcare centers for this study was implemented to secure variation in parental socio-economic status and ethnic background. However, those who agreed to participate in the study may have been individuals with a greater appreciation and awareness of oral health. Thus, caution should be taken in generalising findings of this study to the population of immigrant parents with young children in Bergen.

The present study has some limitations. It might have been biased by under- and over-reporting because of poor recall bias effects as well as illiteracy. Moreover, because the study is based on face-to-face interviews, the responses may be biased due to social desirability. The small numbers of participants, their diverse backgrounds, and various periods of residence in Norway preclude comparisons between groups as well as the impact of acculturation. The one item measuring parental indulgence related to children’ sugar restriction could not adequately capture an indulgent feeding style. Finally, the cross-sectional design limits the ability to draw causal inferences about parental characteristics’ influence on their feeding practice knowledge and attitudes.

### Implications

The present findings point to the need for interventions to support vulnerable immigrant households, since parents in sociodemographically disadvantaged households were found to be more indulgent, presenting with less positive attitudes and less knowledge regarding restricting sugar content in their feeding practices. To encourage healthy dietary habits, parents are certainly in need of culturally appropriate advice and educational sessions provided by health professionals. Collaboration between the dental health services and family primary healthcare centers can improve awareness, promote healthy dietary feeding habits among parents with an immigrant background and consequently improve the oral health of the infants and small children.

## CONCLUSION

Sociodemographically less affluent immigrant parents were more unlikely to report good knowledge, positive attitudes, and weak indulgence related to control of children’s sugar snacking than were their more affluent counterparts. Culturally adapted intervention programs targeting modifiable oral-health risk indicators should be implemented for sociodemographically disadvantaged immigrant parents with special reference to children’s early dietary habits.
